# Uptake of invitations to a lung health check offering low-dose CT lung cancer screening among an ethnically and socioeconomically diverse population at risk of lung cancer in the UK (SUMMIT): a prospective, longitudinal cohort study

**DOI:** 10.1016/S2468-2667(22)00258-4

**Published:** 2023-01-26

**Authors:** Jennifer L Dickson, Helen Hall, Carolyn Horst, Sophie Tisi, Priyam Verghese, Anne-Marie Mullin, Jon Teague, Laura Farrelly, Vicky Bowyer, Kylie Gyertson, Fanta Bojang, Claire Levermore, Tania Anastasiadis, John McCabe, Neal Navani, Arjun Nair, Anand Devaraj, Allan Hackshaw, Samantha L Quaife, Sam M Janes

**Affiliations:** a Lungs for Living Research Centre, UCL Respiratory, University College London, London, UK; b Cancer Research UK and UCL Cancer Trials Centre, University College London, London, UK; c University College London Hospitals NHS Foundation Trust, London, UK; d Tower Hamlets Clinical Commissioning Group, London, UK; e Royal Brompton and Harefield NHS Trust, London, UK; f National Heart and Lung Institute, Imperial College London, London, UK; g Wolfson Institute of Population Health, Queen Mary University of London, London, UK

## Abstract

**Background:**

Lung cancer screening with low-dose CT reduces lung cancer mortality, but screening requires equitable uptake from candidates at high risk of lung cancer across ethnic and socioeconomic groups that are under-represented in clinical studies. We aimed to assess the uptake of invitations to a lung health check offering low-dose CT lung cancer screening in an ethnically and socioeconomically diverse cohort at high risk of lung cancer.

**Methods:**

In this multicentre, prospective, longitudinal cohort study (SUMMIT), individuals aged 55–77 years with a history of smoking in the past 20 years were identified via National Health Service England primary care records at practices in northeast and north-central London, UK, using electronic searches. Eligible individuals were invited by letter to a lung health check offering lung cancer screening at one of four hospital sites, with non-responders re-invited after 4 months. Individuals were excluded if they had dementia or metastatic cancer, were receiving palliative care or were housebound, or declined research participation. The proportion of individuals invited who responded to the lung health check invitation by telephone was used to measure uptake. We used univariable and multivariable logistic regression analyses to estimate associations between uptake of a lung health check invitation and re-invitation of non-responders, adjusted for sex, age, ethnicity, smoking, and deprivation score. This study was registered prospectively with ClinicalTrials.gov, NCT03934866.

**Findings:**

Between March 20 and Dec 12, 2019, the records of 2 333 488 individuals from 251 primary care practices across northeast and north-central London were screened for eligibility; 1 974 919 (84·6%) individuals were outside the eligible age range, 7578 (2·1%) had pre-existing medical conditions, and 11 962 (3·3%) had opted out of particpation in research and thus were not invited. 95 297 individuals were eligible for invitation, of whom 29 545 (31·0%) responded. Due to the COVID-19 pandemic, re-invitation letters were sent to only a subsample of 4594 non-responders, of whom 642 (14·0%) responded. Overall, uptake was lower among men than among women (odds ratio [OR] 0·91 [95% CI 0·88–0·94]; p<0·0001), and higher among older age groups (1·48 [1·42–1·54] among those aged 65–69 years *vs* those aged 55–59 years; p<0·0001), groups with less deprivation (1·89 [1·76–2·04] for the most *vs* the least deprived areas; p<0·0001), individuals of Asian ethnicity (1·14 [1·09–1·20] *vs* White ethnicity; p<0·0001), and individuals who were former smokers (1·89 [1·83–1·95] *vs* current smokers; p<0·0001). When ethnicity was subdivided into 16 groups, uptake was lower among individuals of other White ethnicity than among those with White British ethnicity (0·86 [0·83–0·90]), whereas uptake was higher among Chinese, Indian, and other Asian ethnicities than among those with White British ethnicity (1·33 [1·13–1·56] for Chinese ethnicity; 1·29 [1·19–1·40] for Indian ethnicity; and 1·19 [1·08–1·31] for other Asian ethnicity).

**Interpretation:**

Inviting eligible adults for lung health checks in areas of socioeconomic and ethnic diversity should achieve favourable participation in lung cancer screening overall, but inequalities by smoking, deprivation, and ethnicity persist. Reminder and re-invitation strategies should be used to increase uptake and the equity of response.

**Funding:**

GRAIL.

## Introduction

Lung cancer is the leading cause of cancer death worldwide, accounting for 18·4% of all cancer deaths,^1^ with individuals from lower socioeconomic backgrounds disproportionately affected.^2^, ^3^ Diagnosis at an early stage is key to improving outcomes, owing to the significant disparity in survival for stage I compared with stage IV disease (1-year survival 88% *vs* 19%).^4^ In asymptomatic individuals at increased risk of lung cancer, lung cancer screening using low-dose CT reduces lung cancer mortality due to detection of lung cancer at an earlier stage, when treatments are more effective.^5^, ^6^ In 2014, lung cancer screening using low-dose CT was approved by the United States Preventive Services Task Force (USPSTF) among individuals at high risk of lung cancer (those who were aged 55–80 years, had a smoking history of ≥30 pack-years, and either currently smoke or had quit within the past 15 years),^7^ with other countries implementing pilot programmes.^8^, ^9^

**Research in context**

**Evidence before this study**

We searched Embase, Ovid MEDLINE, and PsychINFO online databases for trials or studies published between Jan 1, 1980 and Feb 18, 2022, reporting the uptake of low-dose screening for lung cancer overall, by smoking status, or by socioeconomic deprivation. No language restrictions were applied. The following search terms were used: “((Lung neoplas* or lung cancer or lung carcinoma or lung adenocarcinoma) and (screen* or mass screening or population screen* or screening program* or early diagnos* or detect* or test*) and (LDCT or low dose CT or low dose computerised tomog* or low dose computed tomog* or CT scan* or spiral CT or chest radio* or chest x-ray or CXR) and (uptake or attend* or particip* or adher* or inequal* or disparit* or complian* or ethnic* or soci* or demograph* or depriv* or divers* or education* or race or racial or sex or gender)).mp. [mp=ti, ab, hw, tn, ot, dm, mf, dv, kf, fx, dq, nm, ox, px, rx, an, ui, sy, tc, id, tm])”. The search retrieved 5334 publications, comprising peer-reviewed papers and conference abstracts. Seven peer-reviewed papers were directly relevant to the presented work, each of which focused on one specific characteristic or ethnic group. The Lung Screen Uptake Trial, on which this study was based, reported the demographic and smoking characteristics of individuals who attended screening. However, this study included a small cohort (n=2012) from a restricted geographical area (north-central and east London) with restricted invitation criteria (individuals aged 60–75 years who had been recorded as smoking by their general practitioner in the previous 7 years), and the aims of the study were to assess the effect of a targeted invitation materials (*vs* usual care invitations) on uptake of a pre-scheduled Lung Health Check appointment offering low-dose CT screening using a randomised controlled trial design.

**Added value of this study**

In the multicentre, prospective, longitudinal SUMMIT study, we report detailed demographic characteristics of individuals who responded and those who did not respond to a lung health check invitation among a socioeconomically and ethnically diverse UK population. Although the overall response was 31%, with a further 14% of individuals responding to re-invitation, those who were male, relatively young, living within areas of higher deprivation, and of Other White ethnicity (ie, of largely European descent other than British) were less likely to respond. Using reminder and re-invitation strategies improved participation among groups less likely to respond to the first invitation, thereby improving the equity of response. Crucially, the SUMMIT study approached individuals on a population basis, mimicked a national organised screening programme, and invited a more diverse population than has been approached previously in the UK. In doing so, this builds on the findings of the Lung Screen Uptake Trial, with participation by ethnicity analysed at a granular level, disaggregated into 16 categories.

**Implications of all the available evidence**

Understanding who does and does not respond to the initial screening invitation provides the foundation for further targeted work on initiatives to increase participation in these under-represented groups. These findings imply that strategies that prompt, remind, and re-invite help to improve equity of response. Further research and initiatives are needed to understand why individuals from other White ethnicities were less likely to respond, using approaches that examine how different population characteristics associated with non-participation might intersect.

The effectiveness and equity of low-dose CT lung cancer screening relies on uptake from groups at high risk of lung cancer, because screening of individuals who are at higher risk improves the risk–benefit ratio. However, uptake has been low, compounded by individuals who are most at risk being less likely to engage in screening—namely, current smokers and people from lower socioeconomic groups.^10^, ^11^, ^12^, ^13^

Comparisons in uptake across different nations are challenging due to differing health-care models and approaches to identify and invite individuals for lung cancer screening. In the USA, where screening is opportunistic and health-care provision is variable, an estimated 14·4% of eligible individuals were screened in 2017–18.^14^ UK programmes have used primary care records to identify and invite potentially eligible individuals for lung cancer screening to a lung health check. Reported uptake with this approach is 20·4–52·6%, with 50·5–84·6% of individuals who respond to the invitation being eligible for lung cancer screening.^15^, ^16^, ^17^ However, none of these programmes accounted for variations in uptake by demographic characteristics.

Disparities in lung cancer screening uptake are observed predominantly by socioeconomic deprivation and smoking status.^11^, ^17^ Although disparities in screening uptake by ethnicity exist for other cancer screening programmes, individuals belonging to individual minority ethnic groups are under-represented in analyses of lung cancer screening programmes, providing scarce data to understand potential disparities.^18^, ^19^ In the few studies worldwide that have reported data on ethnicity and lung cancer screening, more than 90% of participants were White, which is not representative of demographic characteristics of populations eligible for lung cancer screening in the real world;^5^, ^20^, ^21^ however, modelling studies indicate significant benefits of lung cancer screening for individuals from Black ethnic backgrounds.^22^ Additional evidence highlights that there is a paucity of evidence on the true populations eligible for lung cancer screening and uptake of screening invitations, particularly in minority ethnic groups and individuals from areas with greater socioeconomic deprivation.^23^ The population of London (UK) is diverse, with 27·3% of adults aged 55–77 years reporting being from an minority ethnic group.^24^ Although two London-based lung cancer screening studies reported higher proportions of minority ethnic individuals among participants than other studies (16·5% and 15·2%^15^, ^17^), further research is needed to fully understand the representativeness of these data. The low absolute number of people within any individual ethnic group in existing studies has precluded exploration of the association between screening uptake and distinct ethnic groups, beyond aggregated categories of ethnicity.

Variation in uptake by ethnic group is crucial to understand to ensure the disparities observed in lung cancer outcomes for minority ethnic groups and uptake of other screening programmes are not perpetuated.^18^, ^25^ We did a large-scale prospective multicentre study (SUMMIT) that assessed the uptake of an invitation for a low-dose CT lung cancer screening programme in an ethnically diverse UK cohort at high risk of lung cancer as one of the primary outcome measures.

## Methods

### Study design and participants

In this prospective, longitudinal cohort study, individuals aged 55–77 years who were recorded as a current smoker on their National Health Service (NHS) England primary care records any time in the previous 20 years were identified for invitation to a lung health check via electronic record searches (March 20–Dec 12, 2019; appendix p 1). All practices across north-central and northeast London were approached for participation in SUMMIT. 414 agreed to participate in the study and in this study we report results for the first 251 practices where data were extracted. The search excluded individuals with dementia, individuals with metastatic cancer, those receiving palliative care, housebound individuals, and people who declined research participation.

Interested individuals responded by telephone and had their eligibility for a lung health check appointment assessed with telephone screening questions that estimated individual lung cancer risk on the basis of the lung cancer screening eligibility criteria (USPSTF 2014 Low-Dose CT screening criteria and the 2012 Prostate, Lung, Colorectal and Ovarian model [PLCO_m2012_] 6-year lung cancer risk of ≥1·3%).^26^ Further details about telephone screening have been published previously.^26^

Lung health check appointments were at one of four hospital sites in London, UK (University College Hospital, Mile End Hospital, Finchley Memorial Hospital, and King George Hospital where the scanner was a relocatable unit within a hospital carpark). At the lung health check appointment, study eligibility was confirmed with the same USPSTF and PLCO_m2012_ criteria. Individuals who were receiving treatment for an active cancer were excluded, whereas those receiving adjuvant hormonal therapy were included. Eligible individuals were offered low-dose CT screening on the same day or later if more convenient. Current smokers received smoking cessation advice and an opt-out referral to a local smoking cessation service. Very brief advice on smoking cessation was given during the consultation to all current smokers.

Written consent was obtained from participants at the point of determining study eligibility. Ethical approval was obtained from a NHS Research Ethics committee (17/LO/2004) and the NHS Health Research Authority's Confidentiality Advisory Group (18/CAG/0054).

Analysis of secondary outcomes relating to the performance of a multicancer blood test and implementation of low-dose CT screening will be published elsewhere.

### Procedures

We used an evidence-based postal invitation strategy modelled on the Lung Screen Uptake Trial to invite participants to the lung health check.^17^ Invitations for individuals were sent in batches to each primary care practice to manage appointment demand, minimise the impact of national holidays, and ensure appointment availability. A bespoke automated system interacted with primary care electronic records to identify eligible invitees and push mailings to a secure third-party company (Docmail, Bath, UK), which despatched invitations by second-class mail within 24 h.

Two sequences of lung health check invitation letters were sent (appendix p 2). The first included three letters sent at 2-week intervals from the individual's primary care physician: a pre-invitation letter (notifying individuals of the lung health check availability and that they would be invited), an invitation letter, and a reminder letter (sent to individuals who did not respond after ≥2 weeks). The open invitation letters included information about the lung health check and the potential opportunity to have low-dose CT lung cancer screening as part of a study, in addition to an *M.O.T For Your Lungs* leaflet, adapted from the leaflet developed for Lung Screen Uptake Trial.^17^ Letters were in English with a section on the MOT leaflet with contact details in Bengali, Polish, and Turkish. Translators were provided on request and subsequent documents translated where required. Interested individuals were asked to contact a freephone telephone number to find out if they were eligible for a lung health check at which lung cancer screening would be offered.

A second sequence of re-invitation letters (re-invitation letter and reminder letter) was sent to a subgroup of individuals who had not responded (within ≥4 months) to the first sequence. These letters included sentences describing social norms (ie, the number of people in the individual's area participating) based on evidence from colorectal cancer screening,^27^ and were reviewed by patient and public representatives for their readability and acceptability. This cohort was small due to the cessation of invitations and appointments during the COVID-19 pandemic. It was planned that re-invitations would continue to all non-responders; however, this did not happen due to the cessation of invitations and running of the study during the COVID-19 pandemic.

The proportion of individuals invited who responded to the lung health check invitation by telephone was used to measure uptake. This approach was chosen because it is the first active step taken by an individual to participate in lung cancer screening and allowed examination of uptake as a proportion of the total number invited. We also assessed the demographic and smoking characteristics of individuals who responded to the lung health check invitation and re-invitation letters, the type of letter (invitation or reminder) within each sequence that prompted uptake (ie, the stimulus), and the characteristics associated with response to these different letter types.

We extracted demographic and smoking data from primary care records for all invited individuals. Data included age, sex, ethnicity (self-reported by the patient to their primary care practice), last recorded smoking status, and an area-level socioeconomic deprivation rank (Index of Multiple Deprivation [IMD]) converted from residential postcode. Ethnicity was categorised using the EMIS electronic record system, commonly used by primary care practices, into five major groups and 16 subcategories: group 1, White (1a, White British or Mixed British; 1b, White Irish; 1c, other White ethnicity); group 2, Asian (2a, Bangladeshi; 2b, Indian; 2c, Pakistani; 2d, other Asian ethnicity); group 3, Black (3a, African; 3b, Caribbean; 3c, other Black ethnicity); group 4, Mixed (4a, White and Asian; 4b, White and Black African; 4c, White and Black Caribbean; 4d, other mixed ethnicity); group 5, Other (5a, Chinese; 5b, any other ethnicity); group 6, not stated or missing.

### Statistical analysis

Analysis included individuals invited before Dec 31, 2019 to allow time for individuals to respond and minimise the impact of the COVID-19 pandemic on uptake.

The number of people who responded by telephone to the lung health check invitation (for the first sequence of invitation letters and the second sequence of re-invitation letters sent to non-responders only) was expressed as a proportion of the total number invited. Univariate and multivariate logistic regression analyses were used to calculate odds ratios (ORs) and 95% CIs to examine the associations between uptake of the lung health check invitations and re-invitations, and key demographic characteristics (sex, age, ethnicity, IMD quintile, and last recorded smoking status). We also examined associations between uptake and demographic characteristics individually for each of the five broad categories of ethnicity. Additional analyses assessed uptake for each of the 16 distinct ethnic groups compared with those of White British ethnicity.

Data were analysed using SPSS (version 25.0). This study was registered prospectively with ClinicalTrials.gov, NCT03934866.

### Role of the funding source

The funder was involved in study design, but had no role in data analysis, data interpretation, or writing of the report.

## Results

**Figure 1:**
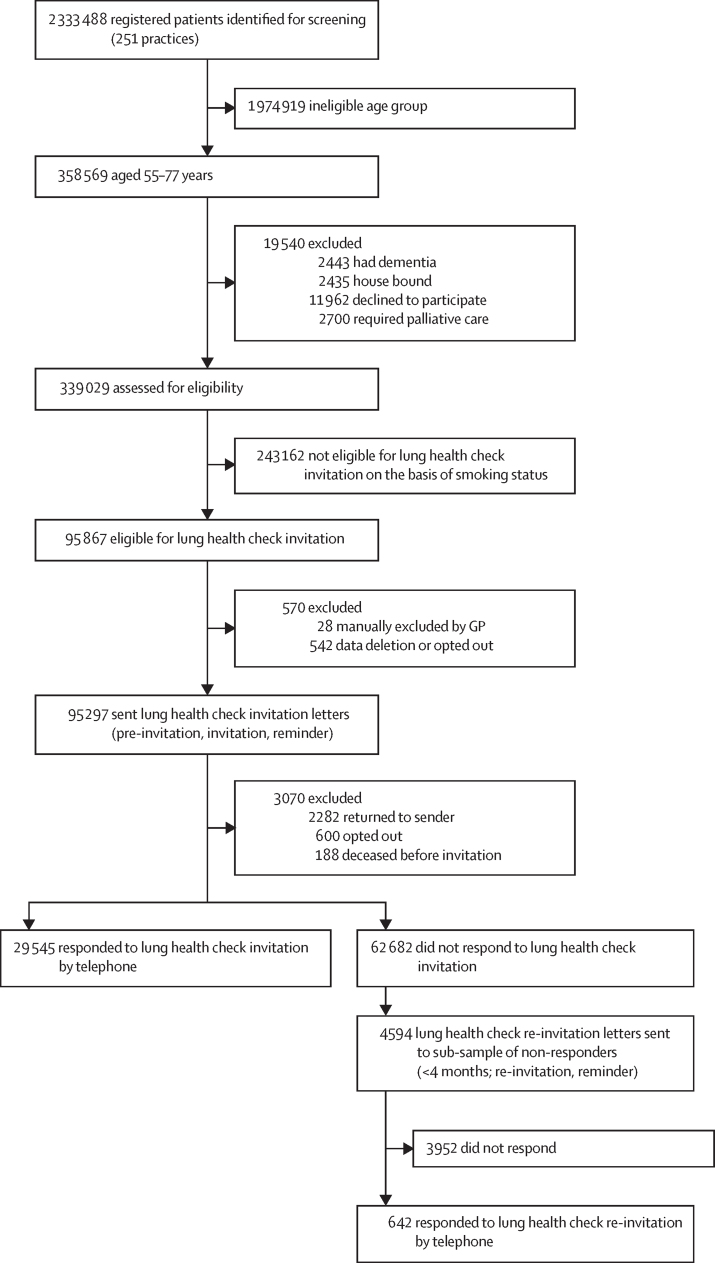
Uptake of the first round of lung health check invitation letters and second round re-invitation letters sent to a subgroup of non-responders GP=general practitioner.

Between March 20 and Dec 12, 2019, the records of 2 333 488 individuals from 251 primary care practices across northeast and north-central London were screened for eligibility (figure 1). Of 358 569 individuals within the eligible age range, 7578 (2·1%) were excluded due to pre-existing medical conditions and 11 962 (3·3%) were not invited since they had opted out of research participation on their primary care records.

In the first sequence, invitation letters were sent to 95 297 individuals between March 25 and Dec 31, 2019 (table 1). The mean age of invited individuals was 63·0 years (SD 6·2), 55 509 (58·2%) invited individuals were male, and 59 886 (62·8%) were from a White ethnic group. 64 246 (67·4%) were categorised as living within the two most deprived IMD quintiles, and 48 518 (50·9%) had been recorded by their primary care physician as a current smoker when last documented. Of 95 297 individuals eligible for invitation, 29 545 (31·0%) responded by telephone.

**Table 1:** Characteristics of invited individuals and individuals who responded to the lung health check invitation

	**All invited (n=95 297)**	**Responded to lung health check invitation (n=29 545)**	**Response (%)**	**Unadjusted OR (95% CI); p value**	**Adjusted OR (95% CI); p value**
**Sex**					
Female	39 787 (41·8%)	12 878 (43·6%)	32·4%	1 (ref)	1 (ref)
Male	55 509 (58·2%)	16 666 (56·4%)	30·0%	0·90 (0·87–0·92); p<0·0001	0·91 (0·88–0·94); p<0·001
Missing	1 (<1·0%)	1 (<1·0%)	..	..	..
**Age, years**					
55–59	34 836 (36·6%)	9108 (30·8%)	26·1%	1 (ref)	1 (ref)
60–64	25 136 (26·2%)	7562 (25·6%)	30·1%	1·22 (1·17–1·26); p<0·0001	1·17 (1·13–1·22); p<0·001
65–69	17 543 (18·4%)	6363 (21·5%)	36·3%	1·61 (1·55–1·67); p<0·0001	1·48 (1·42–1·54); p<0·001
70–75	12 775 (13·4%)	4652 (15·7%)	36·4%	1·62 (1·55–1·69); p<0·0001	1·43 (1·36–1·50); p<0·001
>75	4979 (5·2%)	1833 (6·2%)	36·8%	1·65 (1·55–1·75); p<0·0001	1·41 (1·32–1·50); p<0·001
Missing	28	27	96·4%	..	..
**Ethnicity**					
White	59 886 (62·8%)	18 913 (64·0%)	31·6%	1 (ref)	1 (ref)
Asian	11 690 (12·3%)	3903 (13·2%)	33·4%	1·09 (1·04–1·13); p=0·0001	1·14 (1·09–1·20) p<0·001
Black	8987 (9·4%)	2456 (8·3%)	27·3%	0·82 (0·78–0·86); p<0·0001	0·97 (0·93–1·03); p=0·320
Mixed	1971 (2·1%)	599 (2·0%)	30·4%	0·95 (0·86–1·04); p=0·263	1·07 (0·96–1·18); p=0·217
Other	4821 (5·1%)	1547 (5·2%)	32·1%	1·02 (0·96–1·09); p=0·466	1·09 (1·02–1·16); p=0·011
Not stated	1555 (1·6%)	461 (1·6%)	29·6%	0·91 (0·82–1·02); p=0·105	0·91 (0·81–1·02); p=0·093
Missing	6387 (6·7%)	1666 (5·6%)	26·1%	..	..
**National Index of Multiple Deprivation**					
Quintile 1 (most deprived)	35 300 (37·0%)	9467 (32·0%)	26·8%	1 (ref)	1 (ref)
Quintile 2	28 946 (30·4%)	8652 (29·3%)	29·8%	1·16 (1·12–1·20); p<0·0001	1·14 (1·10–1·18); p<0·001
Quintile 3	15 247 (16·0%)	5193 (17·6%)	34·1%	1·41 (1·35–1·47); p<0·0001	1·37 (1·31–1·43); p<0·001
Quintile 4	11 013 (11·6%)	4255 (14·4%)	38·6%	1·72 (1·64–1·80); p<0·0001	1·65 (1·57–1·73); p<0·001
Quintile 5 (least deprived)	3776 (4·0%)	1597 (5·4%)	42·3%	2·00 (1·87–2·14); p<0·0001	1·89 (1·76–2·04); p<0·001
Missing	1015 (1·1%)	381 (1·3%)	37·5%	..	..
**Last recorded smoking status**					
Current smoker	48 518 (50·9%)	11 685 (39·5%)	24·1%	1 (ref)	1 (ref)
Former smoker	34 145 (35·8%)	13 369 (45·2%)	39·2%	2·03 (1·97–2·09); p<0·0001	1·89 (1·83–1·95); p<0·001
Unknown or other	12 633 (13·3%)	4490 (15·2%)	34·6%	1·74 (1·67–1·81); p<0·0001	1·58 (1·45–1·72); p<0·001
Missing	1 (<1·0%)	1 (<1·0%)	100·0%	..	..

Data are n (%). OR=odds ratio.

Older age groups were more likely than younger age groups to respond to the invitation (OR 1·48 [95% CI 1·42–1·54] among people aged 65–69 years *vs* people aged 55–59 years; table 1). Uptake was lower among men than among women (0·91 [0·88–0·94]), and individuals in the least deprived group were more likely to respond than those in the most deprived groups (1·89 [1·76–2·04]). Former smokers were more likely to respond than current smokers (1·89 [1·83–1·95]).

**Figure 2:**
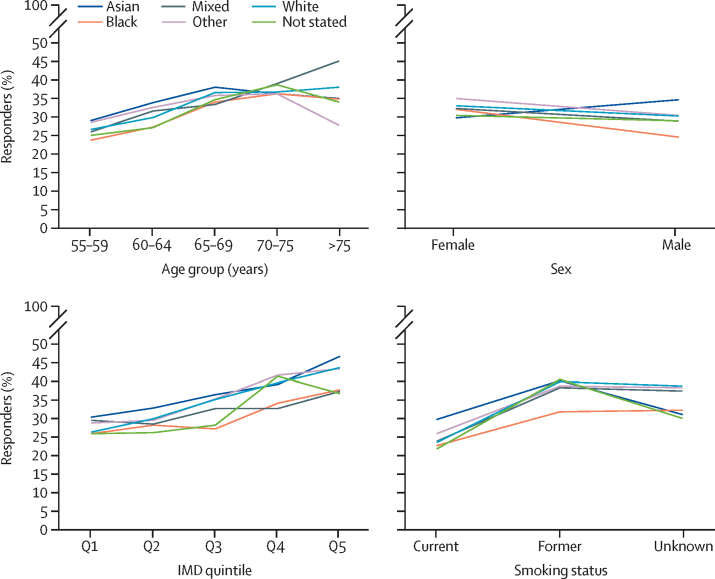
Uptake of the lung health check invitation for each ethnic group stratified by sex, age, deprivation quintile, and smoking status IMD=Index of Multiple Deprivation. Q=quintile.

When ethnicity was analysed as five broad categories, the (unadjusted) uptake of the lung health check invitation varied between ethnic groups and was sometimes influenced by other demographic factors (eg, sex, age, IMD quintile, and smoking status; figure 2).

After adjustment, among White individuals (table 2), those in older age groups (65–75 years and >75 years), those with lower deprivation, and former smokers (*vs* current smokers) were more likely to respond to the invitation, and men were less likely than women to respond to the invitation.

**Table 2:** Uptake of the lung health check invitation for each ethnic group by sex, age, deprivation score, and smoking status

	**White**		**Asian**		**Black**		**Mixed**		**Other**		**Not stated**	
	Responders, n (%)	aOR (95% CI); p value	Responders, n (%)	aOR (95% CI); p value	Responders, n (%)	aOR (95% CI); p value	Responders, n (%)	aOR (95% CI); p value	Responders, n (%)	aOR (95% CI); p value	Responders, n (%)	aOR (95% CI); p value
**Sex**												
Female	9214 (33·1%)	1 (ref)	943 (29·9%)	0·84 (0·77–0·91); p=0·0001	1023 (32·2%)	1·11 (1·02–1·20); p=0·149	279 (32·3%)	1·09 (0·94–1·26); p=0·2765	584 (35·1%)	1·15 (1·03–1·27); p=0·0126	187 (30·5%)	0·89 (0·74–1·06); p=0·01904
Male	9699 (30·3%)	0·89 (0·86–0·92); p<0·0001	2960 (34·7%)	1·14 (1·08–1·20); p<0·0001	1433 (24·7%)	0·80 (0·75–0·85); p<0·0001	9699 (30·3%)	0·93 (0·81–1·07); p=0·3045	963 (30·5%)	0·94 (0·87–1·02); p=0·1425	274 (29·1%)	0·82 (0·71–0·95); p=0·0084
**Age, years**												
55–59	5495 (26·6%)	1 (ref)	1197 (29·1%)	1·17 (1·09–1·27); p<0·0001	1047 (23·7%)	0·95 (0·88–1·02); p=0·1575	237 (26·1%)	1·05 (0·90–1·22); p=0·5451	501 (28·6%)	1·16 (1·04–1·30); p=0·0074	149 (25·0%)	0·92 (0·76–1·12); p=0·4195
60–64	4563 (29·9%)	1·16 (1·11–1·22); p<0·0001	1173 (33·9%)	1·43 (1·32–1·55); p<0·0001	677 (27·3%)	1·13 (1·02–1·24); p=0·0142	174 (31·6%)	1·29 (1·07–1·55); p=0·0072	427 (32·6%)	1·36 (1·21–1·54); p<0·0001	105 (27·1%)	0·97 (0·77–1·22); p=0·7903
65–69	4150 (36·7%)	1·50 (1·43–1·58); p<0·0001	929 (38·1%)	1·64 (1·50–1·79); p<0·0001	395 (34·1%)	1·51 (1·32–1·71); p<0·0001	92 (33·5%)	1·39 (1·07–1·80); p=0·0128	334 (35·9%)	1·53 (1·33–1·76); p<0·0001	92 (34·7%)	1·38 (1·06–1·79); p=0·0163
70–74	3357 (36·8%)	1·44 (1·36–1·52); p<0·0001	422 (36·3%)	1·46 (1·29–1·66); p<0·0001	234 (36·4%)	1·59 (1·35–1·88); p<0·0001	72 (39·1%)	1·64 (1·21–2·22); p=0·0014	223 (36·4%)	1·46 (1·23–1·74); p<0·0001	83 (38·8%)	1·50 (1·13–1·99); p=0·0049
≥75	1330 (38·1%)	1·46 (1·36–1·58); p<0·0001	181 (35·1%)	1·39 (1·15–1·67); p=0·0006	102 (34·9%)	1·49 (1·16–1·90); p=0·0016	23 (45·1%)	2·19 (1·25–3·84); p=0·0061	59 (27·8%)	1·00 (0·74–1·36); p=0·9901	22 (34·0%)	1·14 (0·74–1·76); p=0·5431
**IMD quintile**												
Quintile 1 (most deprived)	5583 (26·4%)	1 (ref)	1419 (30·5%)	1·12 (1·04–1·20); p=0·0016	1286 (26·0%)	1·02 (0·95–1·09); p=0·6495	245 (29·6%)	1·25 (1·07–1·45); p=0·0051	536 (28·9%)	1·38 (1·24–1·54); p<0·0001	115 (26·0%)	1·14 (0·94–1·38); p=0·1833
Quintile 2	3525 (35·3%)	1·18 (1·13–1·24); p<0·0001	1312 (33·0%)	1·28 (1·19–1·38); p<0·0001	815 (28·4%)	1·12 (1·02–1·22); p=0·0125	176 (28·7%)	1·13 (0·95–1·36); p=0·1748	428 (29·6%)	1·39 (1·23–1·57); p<0·0001	128 (26·3%)	1·03 (0·82–1·30); p=0·7791
Quintile 3	3525 (35·3%)	1·44 (1·37–1·52); p<0·0001	635 (36·6%)	1·54 (1·39–1·71); p<0·0001	203 (27·4%)	1·07 (0·91–1·26); p=0·4215	96 (32·9%)	1·37 (1·07–1·75); p=0·0135	271 (35·5%)	1·21 (1·05–1·40); p=0·0078	87 (28·3%)	1·19 (0·92–1·54); p=0·1898
Quintile 4	3014 (39·9%)	1·73 (1·63–1·83); p<0·0001	364 (39·4%)	1·67 (1·46–1·92); p<0·0001	103 (34·3%)	1·46 (1·14–1·85); p=0·0025	55 (32·9%)	1·40 (1·01–1·94); p=0·0428	214 (41·9%)	1·27 (1·07–1·51); p=0·2248	102 (41·6%)	1·39 (1·05–1·84); p=0·0223
Quintile 5 (least deprived)	1135 (43·9%)	1·99 (1·83–2·17) p<0·0001	119 (47·0%)	2·27 (1·76–2·92); p<0·0001	19 (38·0%)	1·69 (0·95–3·00); p=0·0762	18 (37·5%)	1·67 (0·92–3·01); p=0·0899	86 (43·7%)	0·83 (0·61–1·13); p=0·2248	24 (36·9%)	1·08 (0·70–1·67); p=0·7196
**Smoking status**												
Current smoker	7288 (23·7%)	1 (ref)	1501 (29·9%)	1·47 (1·37–1·57); p<0·0001	1066 (22·9%)	1·12 (1·04–1·21); p=0·0026	262 (24·0%)	1·11 (0·96–1·28); p=0·1491	664 (26·1%)	1·18 (1·08–1·30); p=0·0005	175 (21·9%)	0·90 (0·76–1·07); p=0·2407
Former smoker	9281 (40·2%)	2·02 (1·95–2·10); p<0·0001	1370 (40·5%)	2·29 (2·12–2·47); p<0·0001	896 (32·0%)	1·69 (1·55–1·84); p<0·0001	239 (38·5%)	2·07 (1·75–2·44); p<0·0001	629 (38·9%)	2·03 (1·83–2·25); p<0·0001	223 (40·8%)	2·06 (1·73–2·45); p<0·0001
Unknown, not stated, or other	2344 (39·0%)	1·92 (1·81–2·03); p<0·0001	1032 (31·3%)	1·48 (1·36–1·60); p<0·0001	494 (32·4%)	1·69 (1·51–1·89); p<0·0001	98 (37·7%)	1·94 (1·50–2·51); p<0·0001	254 (38·5%)	1·96 (1·67–2·30); p<0·0001	63 (30·3%)	1·25 (0·93–1·70); p=0·1440

ORs were adjusted for gender, age, IMD quintile, and smoking status. aOR=adjusted odds ratio. IMD=Index of Multiple Deprivation.

Overall uptake was higher among individuals with Asian ethnicity than among those with White ethnicity (OR 1·14; 95% CI 1·09–1·20; table 1). Among those with Asian ethnicity, ORs were higher with older age (particularly 65–69 years; 1·64 [1·50–1·79]), among former versus current smokers (2·29 [2·12–2·47]), among those living in areas with lower deprivation (2·27 [1·76–2·92]), and among men versus women (1·14 [95% CI 1·08–1·20]).

Overall, uptake was lower among individuals with Black ethnicity than among those with White ethnicity (OR 0·97 [0·93–1·03]; table 1), with increased uptake among older age groups, individuals living in areas of lower deprivation, and former smokers, and decreased uptake among men (0·80 [0·75–0·85]) compared with women, as observed among White ethnicities. Similar patterns were observed in the other ethnic categories.

When ethnicity was subdivided into 16 groups, the absolute proportion of invited individuals responding to the lung health check invitation varied, ranging from 1195 (38·9%) of 3071 individuals from an Indian ethnic background to 511 (25·8%) of 1977 individuals from an other Black ethnic background (ie, a Black ethnicity other than African or Caribbean; table 3; appendix p 3). Compared with individuals of White British ethnicity, only individuals of other White ethnicity (ie, not British or Irish) were less likely to respond (OR 0·86 [95% CI 0·83–0·90]). Individuals of Chinese, Indian, and other Asian ethnicity were more likely to respond than White individuals (1·33 [1·13–1·56] for Chinese ethnicity; 1·29 [1·19–1·40] for Indian ethnicity; and 1·19 [1·08–1·31] for other Asian ethnicity). Further details of the demographic and smoking characteristics of invited and responding individuals stratified by 16 category ethnic groups are available in the appendix (pp 4–12).

**Table 3:** Uptake of lung health check invitation by ethnicity

	**Invited (n)**	**Responded to lung health check invitation (n)**	**Response (%)**	**Unadjusted OR (95% CI)**	**Adjusted OR (95% CI)**
**Asian or Asian British**					
Bangladeshi or Bangladeshi British	4797	1443	30·1%	0·88 (0·82–0·94); p<0·0001	1·01 (0·94–1·08); p=0·843
Indian or Indian British	3071	1195	38·9%	1·30 (1·21–1·40); p>0·001	1·29 (1·19–1·40); p<0·0001
Pakistani or Pakistani British	1732	515	29·7%	0·86 (0·78–0·96); p=0·006	0·96 (0·86–1·06); p=0·399
Other Asian	2090	750	35·9%	1·14 (1·04–1·25); p=0·004	1·19 (1·08–1·31); p<0·0001
**Black**					
African	2569	729	28·4%	0·81 (0·74–0·88); p<0·0001	0·94 (0·86–1·03); p=0·183
Caribbean	4441	1216	27·4%	0·77 (0·72–0·83); p<0·0001	0·94 (0·88–1·01); p=0·094
Other Black	1977	511	25·8%	0·71 (0·64–0·78); p<0·0001	0·91 (0·82–1·02); p=0·091
**Mixed**					
White and Asian	322	111	34·5%	1·07 (0·85–1·35); p=0·542	1·17 (0·92–1·48); p=0·705
White and Black African	313	84	26·8%	0·75 (0·58–0·96); p=0·024	0·88 (0·68–1·14); p=0·332
White and Black Caribbean	639	179	28·0%	0·8 (0·67–0·95); p=0·010	1·00 (0·84–1·19); p=0·971
Other mixed	697	225	32·3%	0·97 (0·83–1·14); p=0·744	1·06 (0·90–1·25); p=0·488
**Other**					
Chinese	665	254	38·2%	1·26 (1·08–1·48); p=0·004	1·33 (1·13–1·56); p=0·001
Other	4156	1293	31·1%	0·92 (0·86–0·99); p=0·021	1·01 (0·94–1·08); p=0·770
**White**					
British or mixed British	42 522	13 976	32·9%	1 (ref)	1 (ref)
Irish	2860	963	33·7%	1·04 (0·96–1·12); p=0·376	1·06 (0·97–1·15); p=0·191
Other White	14 504	3974	27·4%	0·77 (0·74–0·80); p<0·0001	0·86 (0·83–0·90); p<0·0001
Not stated	1555	461	29·6%	0·86 (0·77–0·96); p=0·008	0·88 (0·79–0·99); p=0·027
Missing	6387	1666	26·1%	..	..

OR=odds ratio.

In the second sequence, re-invitation letters were sent to a subsample of 4594 non-responders (≥4 months after the initial invitation; appendix p 13) between Jan 22 and Feb 10, 2020, on the basis of their primary care practice location (14 difference practices across two Clinical Commissioning Groups [Barnet and Tower Hamlets], which were targeted initially to fit with capacity at the local sites). The demographic characteristics of the subsample were similar to the overall invited sample, although fewer individuals lived within the two most deprived IMD quintiles (2278 [49·6%] of 4594 individuals *vs* 64 246 [67·4%] of 95 297 individuals; appendix p 13).

642 (14·0%) of the 4594 individuals sent a re-invitation letter responded by telephone (figure 1). No independent associations were found for uptake across sex, age group, or ethnic group (analysed as six categories; appendix p 13). Those in the two least deprived groups were significantly more likely to respond than those in the most deprived group (OR 1·73 [95% CI 1·19–2·53], quintile 5 [least deprived] *vs* quintile 1 [most deprived]; 1·46 [1·12–1·89], quintile 4 *vs* quintile 1). Former smokers were more likely to respond than current smokers (OR 1·33 [95% CI 1·10–1·61]).

A greater proportion of invitees responded after receiving the reminder than the invitation letter within the first sequence of lung health check invitation letters (15 746 [53·3%] *vs* 13 585 [46·0%] 29 545) and within the sequence of re-invitation letters sent to the subgroup of non-responders (427 [66·5%] *vs* 215 [33·5%] of 642; appendix p 14). For the initial sequence of lung health check invitation letters, the odds of responding to the reminder letter compared with the invitation letter were significantly increased in Asian (OR 1·27 [95% CI 1·18–1·38]), Black (1·56 [1·42–1·70]), and other (1·26 [1·13–1·40]) ethnic groups, compared with White ethnic groups. Individuals of mixed ethnicity were not more likely to respond to the reminder than the invitation. The odds of responding to the reminder letter rather than the invitation letter significantly decreased with male sex (*vs* female sex; 0·93 [0·88–0·97]), older age (>75 years *vs* 55–59 years; 0·69 [0·62–0·77]), lower deprivation (quintile 5 *vs* quintile 1; 0·79 [0·71–0·89]), and former smoking status (*vs* current; 0·86 [0·81–0·90]).

## Discussion

This study provides demographic data on lung cancer screening uptake from a multicentre population-based lung cancer screening programme offered to a socioeconomically and ethnically diverse population at high risk of lung cancer in the UK. 31% of invited individuals responded to the lung health check invitation, and a further 14% of non-responders responded after being sent re-invitation letters. There was good representation across different demographic subgroups, including people living within the two most deprived quintiles nationally. White backgrounds other than White British (eg, White European), male sex, younger age, deprivation, and current smoking status were all associated with lower uptake.

The response to the lung health check invitation was similar to that in other UK-based lung cancer studies and programmes (range 17·9–52·6%).^15^, ^16^, ^17^ The response rate was improved by re-invitation of non-responders 4 months or more after their first invitation. Uptake was lower than in the Lung Screen Uptake Trial (52·6%)—the study on which the invitation materials were based—and the local average for the NHS bowel cancer screening programme (54·4%), which invites adults of a similar age. Potential reasons for these differences include this being a first-time screening offer, the positioning of lung cancer screening as a research study rather than as a service, and an open booking rather than pre-allocated appointment strategy, which has been shown to increase uptake in other cancer screening programmes, particularly among individuals who did not respond to the initial invitation.^28^, ^29^ Due to the size of the SUMMIT study and the aim of mimicking a service that could be implemented at population scale, scheduling of pre-allocated appointments was not feasible nor cost-efficient, with the telephone eligibility step significantly reducing unused appointments by ineligible invitees.^26^ Although the broad eligibility criteria for invitation via primary care were intended to ensure invitation of all potentially eligible individuals, those deeming themselves ineligible (ie, occasional smoking history) might have chosen to opt out. Overall, the lung health check invitation method provides a feasible and scalable method to identify and invite eligible adults for low-dose CT screening at the population level.

The observed associations between demographic factors and uptake are consistent with the results of previous studies showing persistently lower uptake of lung cancer screening among groups at high risk of lung cancer, including a socioeconomic gradient in uptake and lower uptake among current smokers than among former smokers.^10–13,17^ However, we examined ethnicity in a more granular way than previous studies, using 16 distinct ethnic groups to observe important variations in uptake between people within the same broad ethnic category. Overall, individuals from Asian ethnic backgrounds were more likely to respond than individuals of White ethnicity, which was driven by high response rates among those from Indian and other Asian backgrounds, with uptake from Bangladeshi and Pakistani backgrounds lower in absolute terms. Uptake was also higher among those of a Chinese ethnic background than among those with White British ethnicity. Uptake was significantly lower among those from White ethnic backgrounds other than White British. We show that, in addition to specific ethnic groups, men, younger age groups, those experiencing greater deprivation, and current smokers represent key subgroups to target to improve uptake (table 2).

Previous studies have either homogenised ethnicity into five subgroups^15^, ^20^, ^21^, ^30^, ^31^ or do not report ethnicity at all.^6^, ^16^, ^32^ Our granular findings identify target areas for further research to understand the determinants of ethnic disparities in uptake and highlight the importance of targeted and tailored campaigns to achieve equitable uptake. Inequalities in uptake by ethnicity also exist in UK colorectal and breast cancer screening programmes^18^, ^19^ and targeted interventions will be crucial to pre-empt and mitigate their effects in lung cancer screening programmes.

This study provides strong evidence for implementation of reminder and re-invitation strategies to improve equitable uptake of lung cancer screening. Response was highest for the reminder letter across all recipients, but particularly high among current smokers, people experiencing higher deprivation, and Black and Asian ethnic groups. Repeat invitation of non-responders has been successful in UK breast, cervical, and bowel cancer screening programmes,^33^, ^34^, ^35^ with postal repeat invitation found to be most effective (effect size of approximately 10%).^33^ In this study, the uptake of the lung health check re-invitation to non-responders was higher than that reported in other cancer screening settings (14%). If this approach was applied to all non-responders, it could translate to an additional 9·7% overall uptake, increasing total uptake to 40·7%.

This study benefits from a large, diverse sample, providing sufficient cases to explore uptake of lung cancer screening by 16 distinct ethnic groups. However, the study had important limitations. First, although the extraction of demographic and smoking data from primary care allowed analysis of all invitees, this might reduce the reliability of current smoking status data for invitees who had not visited primary care recently. Second, the interim nature of this analysis meant that uptake data were only analysed for individuals invited before the UK went into national lockdown due to the COVID-19 pandemic, when study sites were temporarily closed. Uptake might be underestimated among individuals invited closer to the national lockdown, including uptake of the re-invitation letters that could only be examined among a subgroup of non-responders, reducing their diversity relative to the overall invited sample. Third, framing the lung cancer screening offer as part of research might have reduced uptake, as invitees might be less inclined to take part in cancer screening offered as research as opposed to a service. Collection of detailed demographic information about lung cancer screening programmes in the service setting will be crucial to ensure that inequalities in uptake are monitored and addressed.

In conclusion, the lung health check approach to invitation via primary care provides a feasible and scalable method to identify and invite individuals to lung cancer screening across a socioeconomically and ethnically diverse population. Reminder and repeat invitation strategies should be adopted to improve overall uptake and reduce inequalities in uptake among individuals at highest risk. Our findings can be used to develop materials targeted to support informed participation among individuals within demographic groups that are less likely to respond to the initial invitation letter. Further research is required to understand the determinants of, and reduce ethnic disparities in, the actual uptake of lung cancer screening.

## Data sharing

Relevant individual de-identified participant data (including data dictionaries) will be made available on reasonable request via e-mail to SMJ (s.janes@ucl.ac.uk) following confirmation by SMJ and the Cancer Research UK and UCL Cancer Trials Centre. Data will be available to share after the publication of the study primary and secondary endpoints.

## Declaration of interests

JLD, CH, ST, HH, and PV were employed by University College London (UCL) as clinical research fellows through SUMMIT Study funding provided by GRAIL. SMJ has received travel expenses from AstraZeneca, BARD1 Bioscience, Optellum, Jansen, Takeda, Evidera, and Achilles Therapeutics for participation on advisory boards; has received honoraria for lectures from Chiesi; and has received travel expenses from AstraZeneca for a US conference. AH has received one honorarium for an advisory board meeting for GRAIL; has received consulting fees from Evidera (for a GRAIL-initiated project); and has previously owned shares in Illumina. NN has received honoraria for advisory, education, and consultancy work from Amgen, AstraZeneca, Bristol-Meyers Squibb, Guardant Health, Janssen, Lilly & Co, Merck Sharp & Dohme, Olympus, Oncimmune, OncLive, PeerVoice, Pfizer, and Takeda, all outside of the submitted work. AN has received consulting fees from Aidence BV, Faculty Science Limited, and MSD; and has received expenses for travelling to a conference from Takeda. A-MM, JT, LF, VB, KG, FB, CL, TA, JM, AD, and SLQ declare no competing interests.
