# Supplementary material for: Uptake of invitations to a lung health check offering low-dose CT lung cancer screening among an ethnically and socioeconomically diverse population at risk of lung cancer in the UK (SUMMIT): a prospective, longitudinal cohort study
Source: Lancet Public Health. 2023 Jan 26;8(2):e130–40. doi: 10.1016/S2468-2667(22)00258-4 (PMC7615156; doi:10.1016/S2468-2667(22)00258-4)
Supplement: Supplementary appendix [file mmc1.pdf]

THE LANCET
Public Health

Supplementary appendix

This appendix formed part of the original submission and has been peer reviewed. We post it as supplied by the authors.

Supplement to: Dickson JL, Hall H, Horst C, et al. Uptake of invitations to a lung health check offering low-dose CT lung cancer screening among an ethnically and socioeconomically diverse population at risk of lung cancer in the UK (SUMMIT): a prospective, longitudinal cohort study. *Lancet Public Health* 2023; **8:** e130–40.

Uptake of invitation to a lung health check for LDCT screening among an ethnically and socioeconomically diverse population at risk of lung cancer in the UK (SUMMIT): a prospective, longitudinal cohort study – Supplementary material

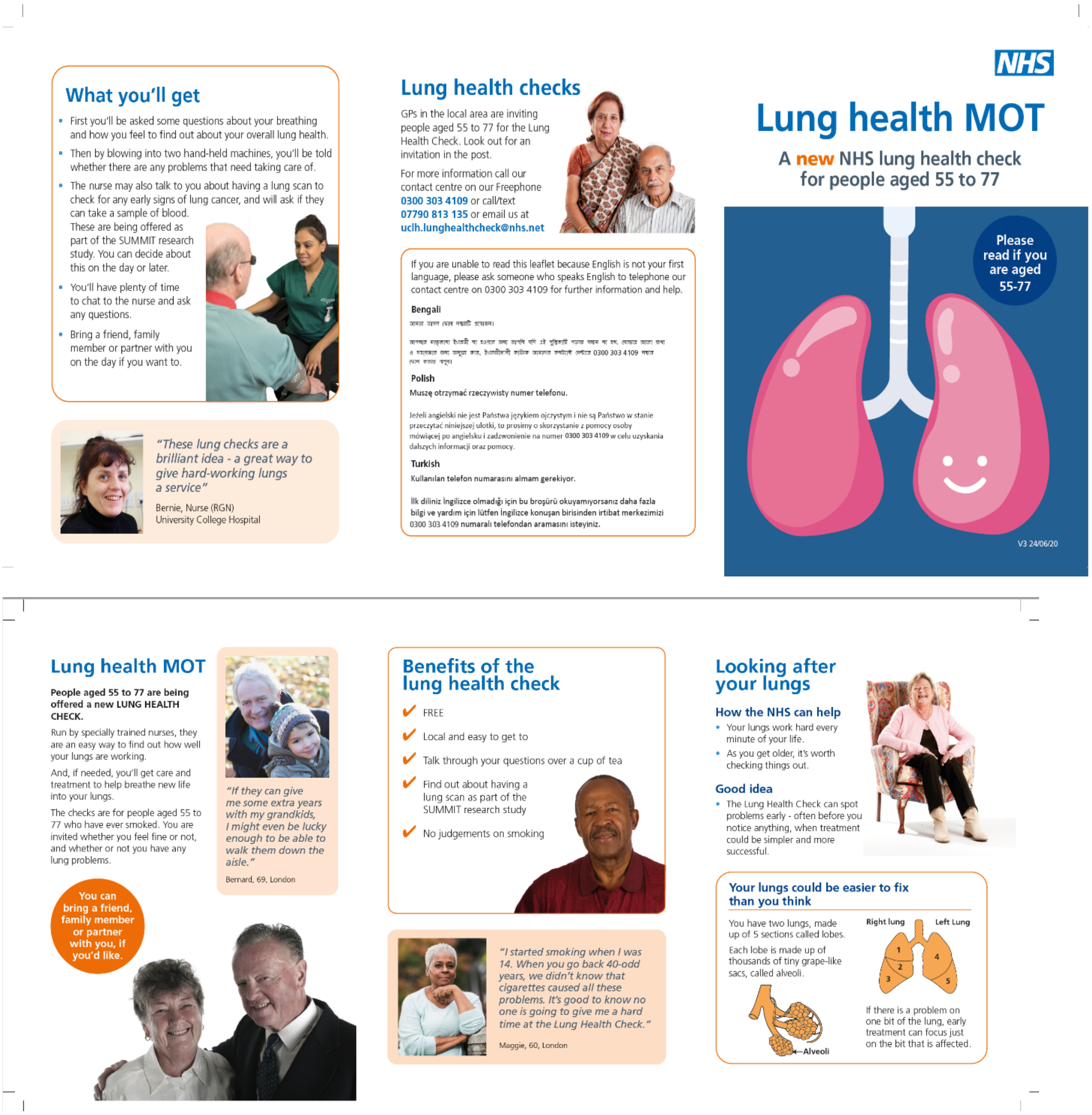

Figure 1: SUMMIT MOT for your lungs leaflet ***(adapted from Lung Screen Uptake Trial and licensed under CC-BY, Quaife et al, 2020, AJRCCM; 201(8): 965-975. DOI:*** 10.1164/rccm.201905-0946OC)

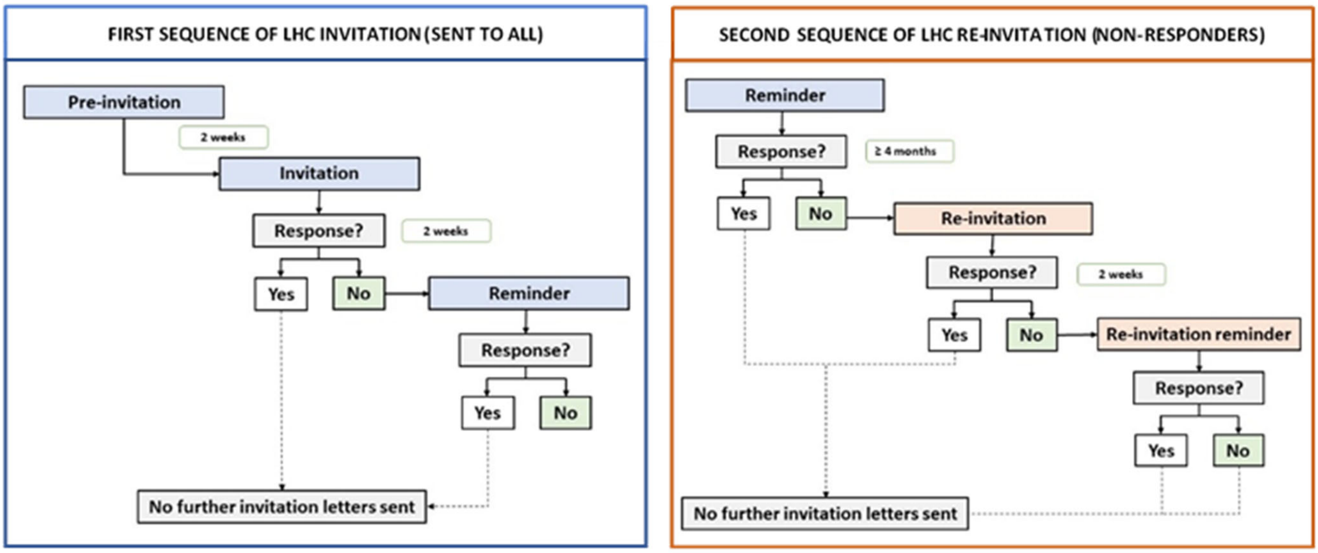

Figure 2: Sequences of LHC invitation letters (sent to all) and LHC re-invitation letters (sent to non-responders >4 months only)

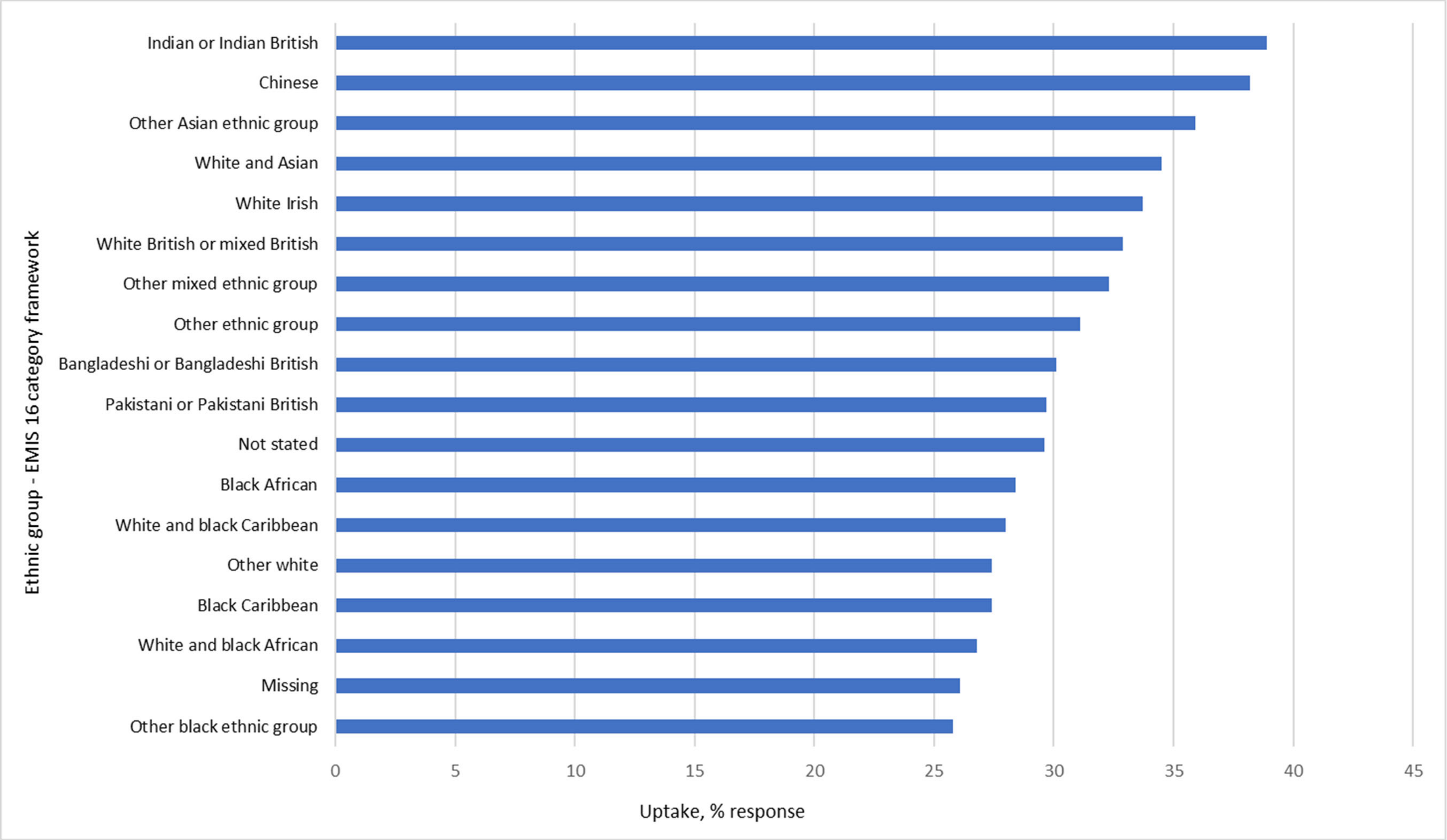

Figure 3: Frequencies for uptake of LHC invitation by 16 categories of ethnicity

Table 1: Frequencies of LHC invitations, responses to invitation and percentage uptake in Asian and Asian British ethnic groups stratified by demographic and smoking characteristics

	Invited (n)	Responded to LHC invitation (n)	% Uptake
**Bangladeshi or Bangladeshi British**			
**Sex (n)**			
**Female**	1796	494	28
**Male**	3001	1027	34
**Age groups, Years (n)**			
**Age 55-59**	1852	584	32
**Age 60-64**	1417	467	33
**Age 65-69**	957	319	33
**Age 70-75**	361	95	26
**Age >75**	210	56	27
**National Index of Multiple Deprivation, (n)**			
**Quintile 1 (most deprived)**	2985	927	31
**Quintile 2**	1314	401	31
**Quintile 3**	338	121	36
**Quintile 4**	113	49	43
**Quintile 5 (least deprived)**	16	10	63
**Last recorded smoking status, (n)**			
**Current smoker**	1944	581	30
**Former smoker**	1166	457	39
**Unknown/other**	1687	483	29
**Indian or Indian British**			
**Sex (n)**			
**Female**	534	198	37
**Male**	2537	1070	42
**Age groups, Years (n)**			
**Age 55-59**	904	286	32
**Age 60-64**	936	404	43
**Age 65-69**	695	337	48
**Age 70-75**	384	175	46
**Age >75**	151	65	43
**National Index of Multiple Deprivation, (n)**			
**Quintile 1 (most deprived)**	591	207	35
**Quintile 2**	1172	464	40
**Quintile 3**	676	303	45
**Quintile 4**	423	194	46
**Quintile 5 (least deprived)**	147	76	52
**Last recorded smoking status, (n)**			
**Current smoker**	1287	454	35
**Former smoker**	1010	486	48
**Unknown/other**	774	328	42
**Pakistani or Pakistani British**			
**Sex (n)**			
**Female**	267	81	30

**Male**	1465	452	31
**Age groups, Years (n)**			
**Age 55-59**	608	160	26
**Age 60-64**	533	155	29
**Age 65-69**	347	128	37
**Age 70-75**	171	66	39
**Age >75**	73	24	33
**National Index of Multiple Deprivation, (n)**			
**Quintile 1 (most deprived)**	509	150	29
**Quintile 2**	822	248	30
**Quintile 3**	269	88	33
**Quintile 4**	98	33	34
**Quintile 5 (least deprived)**	20	9	45
**Last recorded smoking status, (n)**			
**Current smoker**	804	230	29
**Former smoker**	504	174	35
**Unknown/other**	424	129	30
**Other Asian**			
**Sex (n)**			
**Female**	558	223	40
**Male**	1532	581	38
**Age groups, Years (n)**			
**Age 55-59**	752	244	32
**Age 60-64**	572	224	39
**Age 65-69**	438	188	43
**Age 70-75**	246	107	43
**Age >75**	82	41	50
**National Index of Multiple Deprivation, (n)**			
**Quintile 1 (most deprived)**	569	208	37
**Quintile 2**	669	246	37
**Quintile 3**	452	177	39
**Quintile 4**	289	126	44
**Quintile 5 (least deprived)**	70	33	47
**Last recorded smoking status, (n)**			
**Current smoker**	981	344	35
**Former smoker**	701	317	45
**Unknown/other**	408	143	35

The demographic (sex, age and national index of multiple deprivation) and smoking characteristics of individuals categorised as being from Asian ethnic groups including Bangladeshi, Indian, Pakistani, and 'other Asian' as defined by the ONS 16 category criteria.

Table 2: Frequencies of LHC invitations, responses to invitation and percentage uptake in Black ethnic groups stratified by demographic and smoking characteristics

	Invited (n)	Responded to LHC invitation (n)	% Uptake
**African**			
**Sex (n)**			
**Female**	667	218	33
**Male**	1902	539	28
**Age groups, Years (n)**			
**Age 55-59**	1249	312	25
**Age 60-64**	728	214	29
**Age 65-69**	321	127	40
**Age 70-75**	202	78	39
**Age >75**	69	26	38
**National Index of Multiple Deprivation, (n)**			
**Quintile 1 (most deprived)**	1430	406	28
**Quintile 2**	768	230	30
**Quintile 3**	212	62	29
**Quintile 4**	111	37	33
**Quintile 5 (least deprived)**	22	10	45
**Last recorded smoking status, (n)**			
**Current smoker**	1074	265	25
**Former smoker**	854	285	33
**Unknown/other**	641	207	32
**Caribbean**			
**Sex (n)**			
**Female**	1805	605	34
**Male**	2636	641	24
**Age groups, Years (n)**			
**Age 55-59**	2011	485	24
**Age 60-64**	1262	358	28
**Age 65-69**	633	213	34
**Age 70-75**	343	124	36
**Age >75**	191	65	34
**National Index of Multiple Deprivation, (n)**			
**Quintile 1 (most deprived)**	2402	638	27
**Quintile 2**	1504	439	29
**Quintile 3**	344	99	29
**Quintile 4**	129	48	37
**Quintile 5 (least deprived)**	20	6	30
**Last recorded smoking status, (n)**			
**Current smoker**	2459	564	23
**Former smoker**	1364	461	34
**Unknown/other**	618	221	36
**Other black**			
**Sex (n)**			
**Female**	708	230	32
**Male**	1269	302	24
**Age groups, Years (n)**			

**Age 55-59**	1151	282	25
**Age 60-64**	491	132	27
**Age 65-69**	206	67	33
**Age 70-75**	97	39	40
**Age >75**	32	12	38
**National Index of Multiple Deprivation, (n)**			
**Quintile 1 (most deprived)**	1106	279	25
**Quintile 2**	602	169	28
**Quintile 3**	186	55	30
**Quintile 4**	60	21	35
**Quintile 5 (least deprived)**	8	4	50
**Last recorded smoking status, (n)**			
**Current smoker**	1126	267	24
**Former smoker**	586	185	32
**Unknown/other**	265	80	30

The demographic (sex, age and national index of multiple deprivation) and smoking characteristics of individuals categorised as being from Black ethnic groups including African, Caribbean and 'other black' as defined by the ONS 16 category criteria.

Table 3: Frequencies of LHC invitations, responses to invitation and percentage uptake in mixed ethnic groups stratified by demographic and smoking characteristics

	Invited (n)	Responded to LHC invitation (n)	% Uptake
**White and Asian**			
**Sex (n)**			
**Female**	130	45	35
**Male**	192	74	39
**Age groups, Years (n)**			
**Age 55-59**	130	47	36
**Age 60-64**	92	31	34
**Age 65-69**	57	22	39
**Age 70-75**	36	16	44
**Age >75**	7	3	43
**National Index of Multiple Deprivation, (n)**			
**Quintile 1 (most deprived)**	99	35	35
**Quintile 2**	79	31	39
**Quintile 3**	73	23	32
**Quintile 4**	42	20	48
**Quintile 5 (least deprived)**	23	8	35
**Last recorded smoking status, (n)**			
**Current smoker**	178	53	30
**Former smoker**	104	49	47
**Unknown/other**	40	17	43

White and Black African			
**Sex (n)**			
Female	95	29	31
Male	218	56	26
**Age groups, Years (n)**			
Age 55-59	148	34	23
Age 60-64	89	26	29
Age 65-69	48	13	27
Age 70-75	21	9	43
Age >75	7	3	43
**National Index of Multiple Deprivation, (n)**			
Quintile 1 (most deprived)	125	33	26
Quintile 2	108	32	30
Quintile 3	47	11	23
Quintile 4	26	8	31
Quintile 5 (least deprived)	4	0	0
**Last recorded smoking status, (n)**			
Current smoker	170	35	21
Former smoker	87	28	32
Unknown/other	56	22	39
**White and Black Caribbean**			
**Sex (n)**			
Female	317	101	32
Male	322	85	26
**Age groups, Years (n)**			
Age 55-59	316	81	26
Age 60-64	182	55	30
Age 65-69	69	18	26
Age 70-75	51	22	43
Age >75	21	10	48
**National Index of Multiple Deprivation, (n)**			
Quintile 1 (most deprived)	336	94	28
Quintile 2	207	60	29
Quintile 3	61	19	31
Quintile 4	27	10	37
Quintile 5 (least deprived)	5	2	40
**Last recorded smoking status, (n)**			
Current smoker	384	88	23
Former smoker	180	71	39
Unknown/other	75	27	36
**Other mixed**			
**Sex (n)**			
Female	321	119	37
Male	376	119	32
**Age groups, Years (n)**			
Age 55-59	315	88	28
Age 60-64	188	68	36
Age 65-69	101	46	46
Age 70-75	76	28	37

Age >75	16	7	44
**National Index of Multiple Deprivation, (n)**			
Quintile 1 (most deprived)	267	88	33
Quintile 2	220	62	28
Quintile 3	111	50	45
Quintile 4	72	22	31
Quintile 5 (least deprived)	16	9	56
**Last recorded smoking status, (n)**			
Current smoker	358	105	29
Former smoker	250	99	40
Unknown/other	89	34	38

The demographic (sex, age and national index of multiple deprivation) and smoking characteristics of individuals categorised as being from mixed ethnic groups including white and Asian, white and black African, white and black Caribbean and 'other mixed' as defined by the ONS 16 category criteria.

Table 4: Frequencies of LHC invitations, responses to invitation and percentage uptake in 'other' ethnic groups stratified by demographic and smoking characteristics

	Invited (n)	Responded to LHC invitation (n)	% Uptake
**Chinese**			
**Sex (n)**			
Female	145	66	46
Male	520	207	40
**Age groups, Years (n)**			
Age 55-59	236	85	36
Age 60-64	167	75	45
Age 65-69	146	60	41
Age 70-75	95	45	47
Age >75	21	8	38
**National Index of Multiple Deprivation, (n)**			
Quintile 1 (most deprived)	200	76	38
Quintile 2	187	71	38
Quintile 3	150	68	45
Quintile 4	86	40	47
Quintile 5 (least deprived)	26	11	42
**Last recorded smoking status, (n)**			
Current smoker	332	116	35
Former smoker	242	111	46
Unknown/other	91	46	51
**Other**			
**Sex (n)**			
Female	1517	540	36
Male	2639	816	31
**Age groups, Years (n)**			

Age 55-59	1516	442	29
Age 60-64	1144	380	33
Age 65-69	785	291	37
Age 70-75	517	185	36
Age >75	191	55	29
National Index of Multiple Deprivation, (n)			
Quintile 1 (most deprived)	1653	475	29
Quintile 2	1260	372	30
Quintile 3	613	230	38
Quintile 4	425	188	44
Quintile 5 (least deprived)	171	84	49
Last recorded smoking status, (n)			
Current smoker	2214	592	27
Former smoker	1373	546	40
Unknown/other	569	218	38

The demographic (sex, age and national index of multiple deprivation) and smoking characteristics of individuals categorised as being from 'other' ethnic groups including Chinese and 'other' as defined by the ONS 16 category criteria.

Table 5: Frequencies of LHC invitations, responses to invitation and percentage uptake in white ethnic groups stratified by demographic and smoking characteristics

	Invited (n)	Responded to LHC invitation (n)	% Uptake
**British or mixed British**			
Sex (n)			
Female	20145	7104	35
Male	22377	7393	33
Age groups, Years (n)			
Age 55-59	13807	3973	29
Age 60-64	10499	3424	33
Age 65-69	8348	3240	39
Age 70-75	7105	2750	39
Age >75	2746	1093	40
National Index of Multiple Deprivation, (n)			
Quintile 1 (most deprived)	14504	4008	28
Quintile 2	12572	4134	33
Quintile 3	7206	2749	38
Quintile 4	5722	2459	43
Quintile 5 (least deprived)	2057	958	47
Last recorded smoking status, (n)			
Current smoker	21028	5374	26
Former smoker	17363	7329	42
Unknown/other	4131	1794	43
**Irish**			
Sex (n)			

**Female**	1286	475	37
**Male**	1574	533	34
**Age groups, Years (n)**			
**Age 55-59**	752	230	31
**Age 60-64**	663	216	33
**Age 65-69**	637	260	41
**Age 70-75**	576	215	37
**Age >75**	232	87	38
**National Index of Multiple Deprivation, (n)**			
**Quintile 1 (most deprived)**	1106	324	29
**Quintile 2**	935	337	36
**Quintile 3**	468	188	40
**Quintile 4**	277	127	46
**Quintile 5 (least deprived)**	55	23	42
**Last recorded smoking status, (n)**			
**Current smoker**	1429	403	28
**Former smoker**	1181	499	42
**Unknown/other**	250	106	42
**Other white**			
**Sex (n)**			
**Female**	6421	1984	31
**Male**	8083	2183	27
**Age groups, Years (n)**			
**Age 55-59**	6135	1526	25
**Age 60-64**	4097	1112	27
**Age 65-69**	2314	797	34
**Age 70-75**	1443	532	37
**Age >75**	513	198	39
**National Index of Multiple Deprivation, (n)**			
**Quintile 1 (most deprived)**	5555	1428	26
**Quintile 2**	4459	1114	25
**Quintile 3**	2321	753	32
**Quintile 4**	1546	611	40
**Quintile 5 (least deprived)**	472	198	42
**Last recorded smoking status, (n)**			
**Current smoker**	8312	1853	22
**Former smoker**	4568	1771	39
**Unknown/other**	1624	543	33

The demographic (sex, age and national index of multiple deprivation) and smoking characteristics of individuals categorised as being from white ethnic groups including British or mixed British, Irish and ‘other white’ as defined by the ONS 16 category criteria.

Table 6: Frequencies of LHC invitations, responses to invitation and percentage uptake where ethnic group has not been stated stratified by demographic and smoking characteristics

	Invited (n)	Responded to LHC invitation (n)	% Uptake
**Not stated**			
**Sex (n)**			
**Female**	613	193	31
**Male**	942	282	30
**Age groups, Years (n)**			
**Age 55-59**	595	154	26
**Age 60-64**	387	111	29
**Age 65-69**	265	92	35
**Age 70-75**	214	85	40
**Age >75**	94	33	35
**National Index of Multiple Deprivation, (n)**			
**Quintile 1 (most deprived)**	442	119	27
**Quintile 2**	486	132	27
**Quintile 3**	307	91	30
**Quintile 4**	245	104	42
**Quintile 5 (least deprived)**	65	24	37
**Last recorded smoking status, (n)**			
**Current smoker**	800	182	23
**Former smoker**	547	228	42
**Unknown/other**	208	65	31

The demographic (sex, age and national index of multiple deprivation) and smoking characteristics of individuals where ethnic group was not stated.

Table 7: Frequencies and adjusted odds ratios for characteristics of individuals responding to the LHC re-invitation

	All invited (n)	Responded to LHC re-invitation (n)	Response (%)	Adjusted OR (95% CI)
All	4,594	642	13.9	
Sex				
Female	1,954	257	13.2	1.00
Male	2,640	385	14.6	1.13 (0.94-1.36) p=0.195
Missing	0	0	0.0	-
Age groups				
55-59	1,685	214	12.7	1.00
60-64	1,232	172	14.0	1.13 (0.89-1.42) p=0.314
65-69	826	123	14.9	1.17 (0.91-1.51) p=0.228
70-75	631	98	15.5	1.29 (0.98-1.70) p=0.069
>75	220	35	15.9	1.27 (0.85-1.90) p=0.243
Missing	0	0	0.0	-
Ethnicity				
White	2,957	414	14.0	1.00
Asian	593	90	15.2	1.16 (0.89-1.50) p=0.265
Black	258	29	11.2	0.83 (0.55-1.26) p=0.388
Mixed	114	18	15.8	1.22 (0.72-2.09) p=0.459
Other	247	39	15.8	1.13 (0.78-1.63) p=0.535
Not stated	65	7	10.8	0.72 (0.32-1.60) p=0.417
Missing	360	45	12.5	-
National Index of Multiple Deprivation				
Quintile 1 (most deprived)	1,102	138	12.5	1.00
Quintile 2	1,176	146	12.4	0.95 (0.73-1.23) p=0.681
Quintile 3	1,034	144	13.9	1.09 (0.84-1.42) p=0.499
Quintile 4	840	148	17.6	1.46 (1.12-1.89) p=0.005
Quintile 5 (least deprived)	261	49	18.8	1.73 (1.19-2.53) p=0.004
Missing	181	17	9.4	-
Last recorded smoking status				
Current smoker	2,496	305	12.2	1.00
Former smoker	1,527	248	16.2	1.33 (1.10-1.61) p=0.004
Unknown/other	571	89	15.6	1.24 (0.94-1.63) p=0.132
Missing	0	0	0.0	-

Table 8: Frequencies and adjusted odds ratios for characteristics associated with uptake by type of letter for the first round LHC invitation only

	Responded to LHC invitation %, (n)	Responded to LHC reminder %, (n)	Total	Adjusted OR (95%CI) p-value	
All, % (n)	46.4 (13,522)	53.6 (15,622)	29,144*	Of reminder response compared to invitation response	
**Sex, % (n)**					
Female	45.6 (5,794)	54.4 (6,916)	12,710	1.00	-
Male	47.0 (7,727)	53.0 (8,706)	16,433	0.93 (0.88-0.97)	p=0.001
Missing	100.0 (1)	0.0 (0)	1	-	-
**Age, mean (SD)**					
	64.4 (6.2)	63.4 (6.2)	-	-	-
**Age groups, % (n)**					
55-59	40.9 (3,680)	59.1 (5,311)	8,991	1.00	-
60-64	45.5 (3,398)	54.5 (4,067)	7,465	0.84 (0.79-0.90)	p<0.001
65-69	49.9 (3,130)	50.1 (3,139)	6,269	0.74 (0.69-0.79)	p<0.001
70-75	51.5 (2,357)	48.5 (2,223)	4,580	0.71 (0.66-0.77)	p<0.001
>75	51.8 (938)	48.2 (874)	1,812	0.69 (0.62-0.77)	p<0.001
Missing	70.4 (19)	29.6 (8)	27	-	-
**Ethnicity, % (n)**					
White	49.1 (9,135)	50.9 (9,487)	18,622	1.00	-
Asian	42.2 (1,631)	57.8 (2,237)	3,868	1.27 (1.18-1.38)	p<0.001
Black	35.9 (873)	64.1 (1,556)	2,429	1.56 (1.42-1.70)	p<0.001
Mixed	45.8 (272)	54.2 (322)	594	1.06 (0.90-1.25)	p=0.481
Other	43.0 (656)	57.0 (871)	1,527	1.26 (1.13-1.40)	p<0.001
Not stated	44.4 (201)	55.6 (252)	453	1.23 (1.01-1.48)	p=0.036
Missing	45.7 (754)	54.3 (897)	1,651	-	-
**National Index of Multiple Deprivation Rank, % (n)**					
Quintile 1 (most deprived)	43.2 (4,027)	56.8 (5,297)	9,324	1.00	-
Quintile 2	46.0 (3,914)	54.0 (4,603)	8,517	0.92 (0.87-0.98)	p=0.010
Quintile 3	47.7 (2,448)	52.3 (2,687)	5,135	0.91 (0.85-0.98)	p=0.010
Quintile 4	50.6 (2,132)	49.4 (2,078)	4,210	0.82 (0.76-0.89)	p<0.001
Quintile 5 (least deprived)	52.1 (825)	47.9 (760)	1,585	0.79 (0.71-0.89)	p<0.001
Missing	47.2 (176)	52.8 (197)	373	-	-
**Last recorded smoking status, % (n)**					
Current smoker	43.8 (5,036)	56.2 (6,466)	11,502	1.00	-
Former smoker	49.3 (6,505)	50.7 (6,678)	13,183	0.86 (0.81-0.90)	p<0.001
Unknown/other	44.4 (1,980)	55.6 (2,478)	4,458	0.97 (0.90-1.04)	p=0.340
Missing	100.0 (1)	0.0 (0)	1	-	-

NOTE: *401 cases were excluded from these analyses because they did not receive the invitation schedule per protocol (were not sent a reminder letter despite taking > 14 days to respond to the LHC invitation).
